# P-1290. Impact of Infectious Disease Physician Involvement in ICU on Antibiotic De-escalation and Cost Reduction-A Prospective observational study

**DOI:** 10.1093/ofid/ofaf695.1478

**Published:** 2026-01-11

**Authors:** R sanjai, Jaflin Selcia, Shobana Selvaganesan, Shibi Selvaraj, Rithik Dharan

**Affiliations:** Jaya college of paramedical sciences, chennai, Tamil Nadu, India; Jaya college of paramedical sciences, chennai, Tamil Nadu, India; Jaya college of paramedical sciences, chennai, Tamil Nadu, India; Jaya college of paramedical sciences, chennai, Tamil Nadu, India; The Tamil Nadu Dr. MGR Medical University, Chennai, Tamil Nadu, India

## Abstract

**Background:**

This prospective observational study was conducted in the intensive care unit (ICU) of a tertiary care hospital in the month of September and October to evaluate the impact of infectious disease (ID) physician involvement on antibiotic de-escalation and cost reduction. The study population included critically ill patients who were admitted to the ICU over a two -month period and required antibiotic therapy. Patients were divided into two groups: those managed with the direct involvement of an ID physician and those managed without such involvement. Antibiotic costs were calculated by analysing pharmacy records, while clinical data were extracted from EHR (electronic health record). These data were subsequently entered into an Excel spreadsheet and analysed using descriptive statistics.

**Methods:**

Infectious disease (ID) physicians play a crucial role in ICU antibiotic stewardship by guiding appropriate use and de-escalating unnecessary or broad-spectrum regimens. Their involvement helps mitigate antimicrobial resistance, improve patient outcomes, and reduce healthcare costs by curbing the use of expensive antibiotics and minimizing adverse effects. This highlights the significant clinical and economic benefits of ID physician engagement in ICU settings

**Results:**

With ID physician involvement, 38% of patients (76/200) had antibiotic de-escalation compared to 29% (81/278) without, despite fewer patients. Cost reductions were also higher with ID physicians (35.7% vs. 17%). Additionally, ICU stays were shorter (< 7–10 days) with ID physicians but longer ( >7–10 days) without them, emphasizing their role in improving outcomes and efficiency.

**Table:** 

Parameter	With ID physician	Without ID physician
No. of patients	200	278
Antibiotic De-escalation Rate (%)	38%	29%
Cost reduction on their payment due to de-escalation	35.7%	17%
ICU Length of Stay	< 7to10days	>7to10 days

**Conclusion:**

Infectious disease physicians enhance ICU care by optimizing antibiotic use, promoting treatment de-escalation, reducing healthcare costs, and shortening patient stays. This approach helps reduce global antibiotic resistance and ultimately improves patient outcomes

**Disclosures:**

All Authors: No reported disclosures

